# Report of Fractures Treated at the “Buffalo Hospital of the Sisters of Charity,” during the Six Months Ending April 1, 1852, with Practical Remarks

**Published:** 1852-07

**Authors:** Frank H. Hamilton

**Affiliations:** One of the Surgeons


### BUFFALO MEDICAL JOURNAL

AND

### MONTHLY REVIEW.

**VOL. 8.** JULY, 1852. **NO. 2.**

### ORIGINAL COMMUNICATIONS.

---

ART. I. — *Report of Fractures treated at the "Buffalo Hospital of the Sisters of Charity," during the six months ending April* 1, 1852, *with Practical Remarks.* By Frank H. Hamilton, M. D., one of the Surgeons.

*Simple Fracture of the Surgical Neck of the Humerus.* Alexander Ballentine, aged 62 years; admitted Dec. 19, 1851. He had fallen upon the sidewalk, striking upon his right arm. Dr. Johnson, of Buffalo, had reduced the fracture and applied appropriate dressings. No union of the fragments had yet occurred, but as the surfaces were in apposition it was only after considerable manipulation that the crepitus became distinct and gave unequivocal evidence of the fact of a fracture and of its situation.

I have found this a rare fracture; and usually difficult of diagnosis, owing to the nearness of the fracture to the head of the bone, where it is buried beneath the acromion and deltoid. Generally it is not much or at all displaced, and it can then only be recognized by the crepitus. The crepitus is, I think, most readily produced by bending the forearm at a right angle with the arm and making use of the forearm as a lever, rotating the arm on its own axis.

The treatment, after admission, consisted of one gutta percha splint accurately moulded and extending from above the shoulder to below the elbow, and encircling one half of the circumference of the arm. The splint being secured with the usual bandages, &c. The advantage of carrying the splint over the shoulder and below the elbow (the elbow being bent at right angles) and moulding it to the surfaces is, that it is not liable to become displaced upward or downward. One broad splint thus applied is as good, if not better, than two or three narrow splints.

The result is a perfect limb. This has been the result in nearly every case which has come under my notice, whether treated by myself or others. I conclude, therefore, that it is one of the least difficult fractures to treat. Indeed I think the fragments would generally remain in apposition if no apparatus was applied, provided the limb was kept quiet.

*Compound Comminuted Fracture of the Femur.* John Street, aged 18 years. Admitted Jan. 4, 1852. The fractures occurred near the middle of the right femur; one fragment had pushed through the flesh and skin on the back of the thigh.

Assisted by Dr. Hill, of Buffalo, I dressed the fracture with a long straight splint — Potter's splint, a modification of Desault's; also with small side splints and rollers.

On the sixth day, I made an extending gaitre, with adhesive straps and a roller, as recommended lately by Crosby, of Dartmouth, and others.

The wound made by the bone healed promptly, and the case was thus reduced to a simple comminuted fracture. The soreness of the muscles through which the bone was thrust, probably increased their contraction and contributed to the eventual shortening of the limb.

The extension and counter-extension were continued six weeks, and the result has been a union with a shortening of one inch and a quarter. The limb is strong and the halt very slight.

I am scarcely satisfied with the adhesive plaster — for although no excoriations have been produced, yet it has been constantly yielding and requiring readjustment or retightening. The cotton and paste-bandage gaitre, as heretofore used by me and described at page 140 of the 5th vol. of this Journal, neither produces excoriations nor requires readjustment.

*Transverse Fracture of the Patella.* Edmund Laffy, aged 20 years, was admitted Oct. 30, 1851. The fracture occurred six days before; Dr. Shaw, of Silver Creek, had dressed it with Sir Astley Cooper's straps, rollers, &c. The same apparatus was continued with but slight modification until the 26th of Nov., when I removed the Cooper dressing and applied a straight splint, with adhesive straps, thus:

First, the splint — A single inclined plane, extending from the ischium to beyond the foot, the lower extremity of which was six inches higher than the upper; the splint was ten inches wide at the ischium and gradually narrowing to six inches at the foot, and furnished with an upright foot board. The splint thus constructed, being cushioned with a well made and carefully adjusted junk, the limb was laid upon it. Beneath the ham the padding was sufficient to raise the knee slightly — a position which I have always found less painful to the patient than complete extension.

Second. The limb was slightly secured to the splint by several strips of bandage.

Third. The upper fragment of the patella was now brought to its place while the center of a broad and long strip of adhesive plaster was made to embrace it from above, and to pass around the sides of the splint until they met and crossed behind and at a point below the knee. A similar strip was now applied below and upon the lower fragment, crossing behind and above. After which several other pieces were made to cover the patella more completely to prevent the fragments from becoming displaced forward, and to prevent also œdema of the knee.

The apparatus remained undisturbed seventeen days, during which it did not become loosened or disarranged — nor was it in any degree painful.

When removed, the patella had formed a very close and satisfactory ligamentous union.

I have always applied my bandages for securing the fragments in apposition, over the leg and *splint*, so as to obviate ligation of the limb; and for the same purpose I have made the splint broad, but I have never before in this fracture used the adhesive straps. I was charmed, as were also my pupils, with their convenience and efficiency.

*Simple Oblique Fracture of Tibia and Fibula.* Walter Redmond, aged 38 years; healthy; broke left leg below the middle, Dec. 13, 1850, by a misstep while walking in the street. On the 15th he was admitted to the hospital, and on the 17th I applied the paste bandage in the usual manner, and then laid the leg and thigh over a double-inclined place.

Dec. 19. Opened the dressing at one point to examine the limb.

Dec. 20. Opened the dressing from end to end, and as, in consequence of the subsidence of swelling and drying of the bandages, they had become loose, I cut off a strip through the whole length, so as to reduce the circumference of the splint, and then closed it again with ten or twelve pieces of tape. From this time I examined the limb frequently until the 19th of Jan., when the dressings were finally removed.

The limb was never but slightly painful; no excoriations occurred; he walked on crutches some days before the splints were taken off, and every thing was removed thirty-three days after the fracture. The limb is neither shortened or bent. The callus was very slight.

*Simple Fracture of Tibia and Fibula.* John Maloney, aged 16 years, broke his left leg in the lower third, while wrestling, and was admitted to the hospital on the next day, Jan. 8, 1852. The limb had been dressed by Dr. Lockwood, of Buffalo, with side splints roller, &c. As every thing appeared snug and in order, the dressings were not removed for the purpose of examining the limb, until the 28th, or three weeks from the time of their application. The fragments were then found in exact apposition.

The dressings were finally removed on the 7th of February, four weeks and a half from the time of fracture. The union was then complete, but he was not allowed to leave his bed for several days.

This case illustrates the advantage of so neatly adjusting the bandages, &c., at the first dressing that a renewal will not be necessary for a length of time; perhaps not until the union is nearly perfected. The union has been more rapid, and probably more perfect, in consequence.

The following cases having been treated as fractures only temporarily, will not be reported in detail:

*Fracture of Os Innominatum and Neck of Femur.* John O'Keife, admitted Oct. 23, 1851. Terminated fatally in a few hours.

*Fracture of Inferior Maxilla and Superior Ossa Nasi, etc., etc.* John Gallahan, admitted Oct. 3, 1851. Terminated fatally in twelve days.

*Compound Fracture of Tibia and Fibula.* Admitted March 25th, 1852. The leg was amputated soon after.

